# Alzheimer’s Disease-Related Metabolic Pattern in Diverse Forms of Neurodegenerative Diseases

**DOI:** 10.3390/diagnostics11112023

**Published:** 2021-11-01

**Authors:** Angus Lau, Iman Beheshti, Mandana Modirrousta, Tiffany A. Kolesar, Andrew L. Goertzen, Ji Hyun Ko

**Affiliations:** 1 Department of Human Anatomy and Cell Science, University of Manitoba, Winnipeg, MB R3E 0J9, Canada; laua1@myumanitoba.ca (A.L.); Iman.beheshti@umanitoba.ca (I.B.); Tiffany.Kolesar@umanitoba.ca (T.A.K.); 2 Neuroscience Research Program, Kleysen Institute for Advanced Medicine, Winnipeg, MB R3E 0Z3, Canada; 3 Undergraduate Medical Education, University of Manitoba, Winnipeg, MB R3E 3P5, Canada; 4 Department of Psychiatry, University of Manitoba, Winnipeg, MB R3E 3N4, Canada; mmodirrousta@sbgh.mb.ca; 5 Department of Radiology, University of Manitoba, Winnipeg, MB R3T 2N2, Canada; andrew.goertzen@umanitoba.ca; 6 Graduate Program in Biomedical Engineering, University of Manitoba, Winnipeg, MB R3E 5V6, Canada

**Keywords:** FDG-PET, machine learning, support vector machine, metabolic classification, neurodegenerative disease, dementia, Alzheimer’s disease, frontotemporal lobar degeneration, dementia with Lewy bodies, biomarker

## Abstract

Dementia is broadly characterized by cognitive and psychological dysfunction that significantly impairs daily functioning. Dementia has many causes including Alzheimer’s disease (AD), dementia with Lewy bodies (DLB), and frontotemporal lobar degeneration (FTLD). Detection and differential diagnosis in the early stages of dementia remains challenging. Fueled by AD Neuroimaging Initiatives (ADNI) (Data used in preparation of this article were obtained from the Alzheimer’s Disease Neuroimaging Initiative (ADNI) database. As such, the investigators within ADNI contributed to the design and implementation of ADNI and/or provided data but did not participate in analysis or writing of this report.), a number of neuroimaging biomarkers for AD have been proposed, yet it remains to be seen whether these markers are also sensitive to other types of dementia. We assessed AD-related metabolic patterns in 27 patients with diverse forms of dementia (five had probable/possible AD while others had atypical cases) and 20 non-demented individuals. All participants had positron emission tomography (PET) scans on file. We used a pre-trained machine learning-based AD designation (MAD) framework to investigate the AD-related metabolic pattern among the participants under study. The MAD algorithm showed a sensitivity of 0.67 and specificity of 0.90 for distinguishing dementia patients from non-dementia participants. A total of 18/27 dementia patients and 2/20 non-dementia patients were identified as having AD-like patterns of metabolism. These results highlight that many underlying causes of dementia have similar hypometabolic pattern as AD and this similarity is an interesting avenue for future research.

## 1. Introduction

Major neurocognitive disorder, commonly known as “dementia” is a broad term used to describe cognitive dysfunction including deficits in memory, self-management, language skills, problem solving, attention, and/or visual perception [1,2]. Psychological changes such as apathy, anxiety, depression, irritability, psychotic symptoms, sleep issues, hallucinations, inappropriate behavior, and paranoia are also common, depending on the underlying disease [1,3]. Importantly, for these symptoms to be classified as dementia, the patient must experience a dramatic impairment to their normal daily life and activities [2,4]. If cognitive dysfunction is mildly to moderately interfering with day-to-day life, this is classified as mild neurocognitive disorder or mild cognitive impairment (MCI) [1]. The number of people suffering from dementia is estimated to be 66 million by 2030 [5], which is estimated to significantly inflate the societal and financial burden (currently in the hundreds of billions of USD annually) [6].

Because dementia has such a broad range of causes, it has recently been categorized as either neurodegenerative (previously “irreversible”) and non-neurodegenerative (previously “possibly reversible”) in origin [2]. Overall, approximately 60–70% of dementia cases are attributable to Alzheimer’s disease (AD)—a neurodegenerative disease with the majority of cases occurring after the age of 65 [2]. After AD, the most common neurodegenerative causes of dementia are dementia with Lewy bodies (DLB) and frontotemporal lobar degeneration (FTLD). DLB is characterized by aggregation of alpha-synuclein proteins in the brain—these same misfolded proteins as also found in Parkinson’s disease (PD) [2]. FTLD typically has a mean disease onset age in a patient’s 60s and is characterized by frontal and temporal lobe atrophy associated with tau and ubiquitin proteins [1]. FTLD can be one of two subtypes: behavioural (changes in personality/behaviour; bvFTLD) or language (semantic dementia, progressive nonfluent aphasia, or logopenic aphasia subtypes, also collectively referred to as primary progressive aphasia (PPA)) [1]. Mixed dementia is also common, especially as patients age, and has several underlying causes, for example, mixed dementia can include a combination of AD, vascular dementia, or DLB [2,7].

Non-neurodegenerative causes of dementia include vascular dementia (e.g., due to stroke, ischemic encephalopathy) which causes as high as 15–20% of dementia cases [8], metabolic disorders, medication, head trauma (e.g., subdural hematomas and diffuse axonal injury), and infection (e.g., human immunodeficiency virus) [2]. Additionally, there are several disorders or diseases that may mimic dementia, but may not develop into full dementia, such as MCI and primary psychiatric disorder [2]. Table 1 presents a brief overview of some of the most common diagnoses associated with dementia, as well as a brief summary of neuroanatomical findings, including findings from fluorodeoxyglucose positron emission tomography (FDG-PET) data. For more information related to the role of FDG-PET in neurodegenerative diseases, see [9,10,11].

The underlying cause of dementia can be difficult to determine based on similar symptom profiles, especially in early stages, while the underlying pathophysiology further complicates diagnosis. For example, amyloidogenic proteins such as tau proteins and amyloid plaques can be involved in different disease processes. Tau proteins accumulate to form neurofibrillary tangles which are found in both FTLD and AD [34], while beta-amyloid forms the amyloid plaques characteristic of AD. Higher than normal concentrations of both tau proteins and beta-amyloid are also found in PD dementia and DLB [35]. Currently, a neurodegenerative disorder-related dementia diagnosis is made predominantly on clinical observation and patient interviews. These include a thorough clinical history, neurological examination assessing mental status, blood samples to rule out metabolic and vitamin abnormalities, and a structural brain scan [2]. Although neuroimaging and genetic testing (e.g., Apolipoprotein E (APOE) which increases the risk of AD) can aid early diagnosis, quantitative biomarkers are still lacking. With the exception of rare genetic forms of disease, the gold standard for confirming a diagnosis requires post-mortem histopathological analysis. In addition to being able to provide more accurate prognostication for patients, accurate diagnoses will become increasingly important as disease modifying therapies targeting various disease pathways are developed. Additionally, successful interventions may only be effective in the early stages of disease, before widespread, irreversible damage and cell death has occurred [36]. These factors emphasize the importance of research on the development of accurate diagnostic tools that are able to discriminate between diseases and identify them early.

Collaborative efforts, led by Dr. Weiner at the AD Neuroimaging Initiative (ADNI; http://www.adni-info.org; accessed on 15 March 2015) have led to the collection of more than 13,000 scans from 2500 participants, acquired from over 50 sites across North America. Utilizing the ADNI database, a number of studies constructed quantitative biomarkers that may aid AD diagnosis [37,38,39,40,41,42,43,44,45]. However, it is yet unclear if these AD-based neuroimaging biomarkers are specific to AD and thus raise questions of whether the use of neuroimaging biomarkers will be helpful in clinics.

Our group previously developed a Machine learning-based Alzheimer’s disease Designation (MAD) algorithm using the FDG-PET data collected by ADNI [44]. FDG is the most commonly used radiotracer for PET, aiding dementia diagnosis. The MAD algorithm provides a quantitative binary outcome, indicating whether or not the spatial topography of a given FDG-PET image matches the characteristic FDG-PET pattern of AD patients. In our previous retrospective study, interestingly, patients with other neurodegenerative diseases with aspects of dementia, such as PD dementia or DLB, were also classified as having the AD-related FDG-PET pattern, whereas cognitively healthy patients with PD were not [44]. These findings suggest that there may be similarities in the brain glucose metabolic pattern among patients with different types of dementia. In the present retrospective study, we investigate how the MAD program classifies different patient groups seen in our local neuropsychiatric clinic and further demonstrates that a common phenotype of glucose metabolic pattern exists across the spectrum of dementia.

## 2. Materials and Methods

All FDG-PET images were obtained from the neuropsychiatric clinic (MM) at St. Boniface Hospital in Winnipeg, Manitoba, Canada, and were retrieved from 2015 to 2019. These patients were referred for FDG-PET because they presented neuropsychiatric symptoms that do not meet typical characteristics of a single disease category. Participants underwent metabolic imaging with FDG-PET after fasting for at least 6 h before scanning. Patients were injected with i.v. [^18^F]-FDG (185 MBq) and a 15-min static image was acquired starting 40 min post-injection. A head computed tomography (CT) scan was acquired for attenuation correction purposes. All PET imaging data for this project were acquired on a Siemens Biograph 16 HiRez PET/CT scanner (Siemens Medical Solutions, Knoxville, TN, USA), located in the John Buhler Research Centre at the Bannatyne campus of the University of Manitoba. Clinical charts were reviewed in August 2020. Image and chart retrieval were conducted with ethics approval (approval number: HS18972, approved on 1 September 2015) from the Biomedical Research Ethics Board at the University of Manitoba, Health Sciences Centre, and St. Boniface hospital and in compliance with the patient health information act (PHIA).

Participants were classified based on their most recent clinical diagnoses (average follow-up duration after PET scans: 13.10 ± 15.49 months), such as the Montreal Cognitive Assessment (MoCA), Mini Mental Status Exam (MMSE), and Frontal Assessment Battery (FAB). The groups used were dementia not yet differentiated, AD, DLB, FTLD, MCI, primary psychiatric disorder, and cognitively healthy (CH; see Table 2). In this study, we excluded data from participants that did not put sufficient effort for allowing clinical diagnoses to be made (*n* = 1). As our primary goal was to explore the signature of dementia in neurodegenerative diseases, we divided our dataset into dementia (*n* = 27, mean age ± SD: 63.25 years ± 8.32, age range: 49–80 years, females: 33%) and non-dementia (*n* = 20, mean age ± SD: 56.00 years ± 10.04, age range: 41–78 years, females: 40%) cohorts. There was a significant difference between dementia and non-dementia cohorts in terms of age at examination (*t*(45) = 2.70, *p* = 0.01). Table 2 presents the patients’ demographic data.

All FDG-PET images were pre-processed and analysed as described elsewhere [44]. Briefly, FDG-PET images were spatially normalized to the PET template provided by SPM12 software (https://www.fil.ion.ucl.ac.uk/spm/; accessed on 1 March 2015) using the “old Normalize” routine as this does not require a T1w image for normalization. Data were then smoothed with a Gaussian filter (kernel size: 8 mm × 8 mm × 8 mm). To compute the participant scores from their PET data, we used our pre-trained MAD framework (https://www.kolabneuro.com/software1; accessed on 1 March 2015). The details of MAD derivation and performance validation have been described elsewhere [44]. In summary, the MAD framework was trained on the basis of metabolic brain features of 94 AD patients and 111 age-matched CH controls [44]. Among the five different prediction algorithms that were tested, support vector machine–iterative single data algorithm (SVM-ISDA; no assumption in the initial estimates, alpha; kernel offset parameter = 0.1; kernel scale = 1; misclassification cost = [0 1; 1 0]; maximal number of numerical optimization iterations = 1,000,000; Feasibility gap tolerance = 0; tolerance for gradient difference = 0) showed the best performance in predicting AD vs. non-AD (i.e., a sensitivity of 0.84 and specificity of 0.95, by 10-fold cross-validation) [44], and thus we selected this model as our main classifier.

## 3. Results

Forty-seven patients were scanned with FDG-PET between 2015 and 2019. The summary of results for all patients are presented in Table 3. More than 66% of the patients with different subtypes of dementia showed AD-like brain metabolic patterns using MAD (18/27), whereas this rate in the non-dementia group was only 10% (2/20). Thus, the sensitivity and specificity for differentiating dementia from non-dementia patients in neurodegenerative diseases was 67% and 90%, respectively.

**Figure 1:**
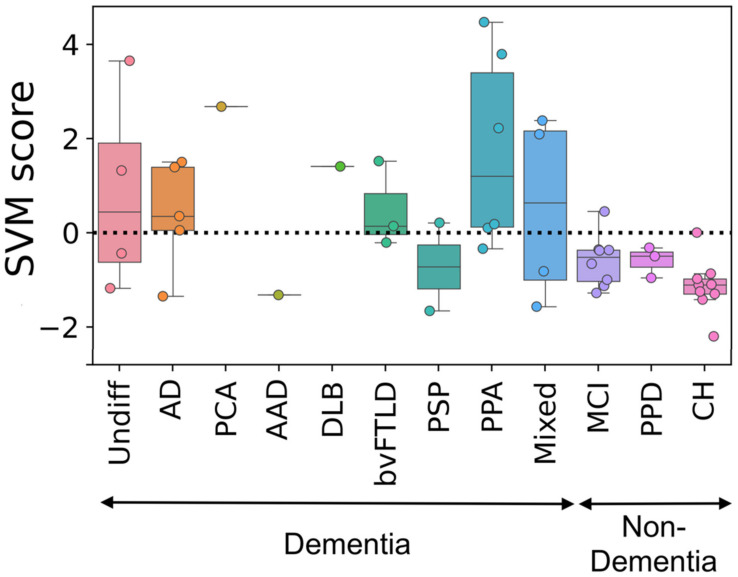
SVM scores generated by MAD in different test groups. A positive score means that MAD classified the subject’s FDG-PET brain image as similar to AD patients’ images. Undiff: Undifferentiated dementia; AD: Alzheimer’s disease; PCA: Posterior cortical atrophy; AAD: Atypical Alzheimer’s disease (frontal); DLB: Dementia with Lewy bodies; bvFTLD: Frontotemporal lobar degeneration, behavioural variant; PSP: Progressive supranuclear palsy; PPA: Primary progressive aphasia; Mixed: Mixed dementia (vascular + AD); MCI: Mild cognitive impairment; PPD: Primary psychiatric disorder; CH: Cognitively healthy.

**Figure 2:**
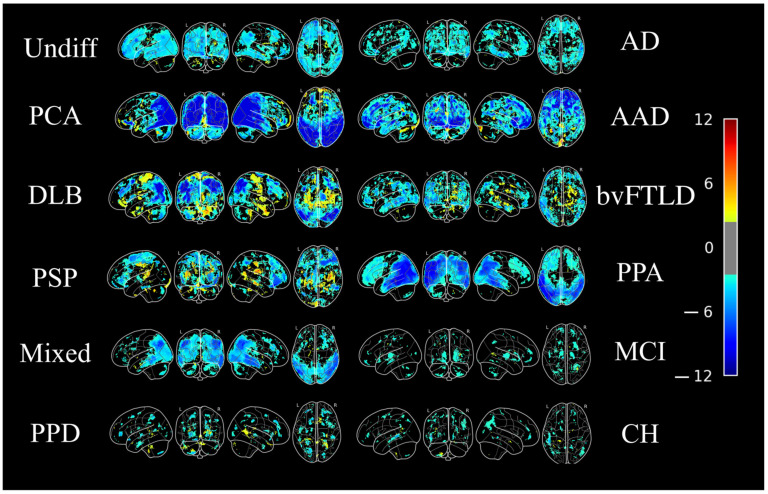
Averaged FDG-PET brain images of different types of dementia. The FDG-PET images have been z-scored to the mean and standard deviation of 111 cognitively healthy individuals that were included in the training set of MAD derivation in our previous study [44], then averaged within each dementia type. Undiff: Undifferentiated dementia; AD: Alzheimer’s disease; PCA: Posterior cortical atrophy; AAD: Atypical Alzheimer’s disease (frontal); DLB: Dementia with Lewy bodies; bvFTLD: Frontotemporal lobar degeneration, behavioural variant; PSP: Progressive supranuclear palsy; PPA: Primary progressive aphasia; Mixed: Mixed dementia (vascular + AD); MCI: Mild cognitive impairment; PPD: Primary psychiatric disorder; CH: Cognitively healthy. The colour bar represents *z* value.

Five patients were clinically diagnosed with AD in the follow-up visits, four of which were accurately classified as AD by MAD. The majority of patients with other subtypes of dementia such as posterior cortical atrophy (1/1), DLB (1/1), bvFTLD (2/3), PSP-F (1/1), and PPA (5/6) groups were also classified as AD. Indeed, the patients in these groups exhibit the AD-like brain metabolic patterns. Two patients (out of four) in the undifferentiated dementia group were identified as AD. In the non-dementia cohort, 7/8 participants in the MCI group, 3/3 in the primary psychiatric disorder group, and 8/9 in the CH group were classified as non-AD. The boxplots of the SVM values generated by MAD in different test groups is shown in Figure 1, whereas Figure 2 displays the projection of z-maps in different types of neurodegenerative diseases compared to the CH group in the training set from our previous work (*N* = 111) [44].

## 4. Discussion

In this study, we used a robust prediction framework with desirable performance for identifying an AD-related metabolic pattern [44]. To our knowledge, this is the first study that explored the AD-related metabolic patterns in different types of neurodegenerative diseases, with respect to dementia. The majority of participants in the dementia cohort (18 out of 27) were labelled as having an AD-related brain metabolic pattern when using the MAD algorithm described in [44]; the majority of participants in the non-dementia cohort (18 out of 20) were identified as non-AD. Our MAD framework showed a desirable performance for distinguishing dementia patients from non-dementia participants (sensitivity: 67%, specificity: 90%) in the present sample. Although there was a significant difference in age at examination between dementia and non-dementia cohorts, it is worth noting that age was not associated with an SVM-ISDA designation of AD [44].

Most of those in the AD group (4/5) were correctly categorized as having AD, in line with our previous work [44]. We further confirmed and extended the findings in our previous work showing that the MAD algorithm also classifies brain metabolic patterns of other underlying causes of dementia as AD-like in nature [44]. Indeed, there is considerable overlap in regions of hypometabolism between AD and various other causes of dementia (see Table 1) [9,14,15,18,19,22,23,46]. For instance, AD and FTLD share frontal and temporal lobe hypometabolism; however, in FTLD this hypometabolism extends to more anterior regions of these lobes, in addition to hypometabolism in the anterior cingulate cortex; metabolism in these regions is typically preserved with the exception of mild hypometabolism of the frontal lobe in AD [47]. On the other hand, AD tends to exhibit hypometabolism in the bilateral posterior parietotemporal lobe and the posterior cingulate cortex while these are preserved in the early stages of FTLD [47]. Further complicating differential diagnoses of dementia, DLB and PCA also show hypometabolism in bilateral posterior parietotemporal and posterior cingulate regions. These diseases additionally show hypometabolism in medial (DLB) and lateral (PCA) occipital cortex, which are preserved in AD and FTLD [47].

Given the literature indicating metabolic similarities between AD and various other dementia-causing neurodegenerative diseases, it is not surprising that many of these diseases were positively identified as having AD-like metabolism by the MAD algorithm. These results highlight that while the MAD algorithm did not readily distinguish between AD and other causes of dementia, it did distinguish between dementia and the potential precursor for dementia (i.e., MCI which had 1/8 MAD-positive) and primary psychiatric disorders (0/3 MAD-positive), which can mimic dementia symptoms.

Many of the large neuroimaging databases such as the ADNI are focused on single diseases and their precursors (e.g., MCI), but have not yet considered other causes of dementia. Thus, little emphasis has been placed on identifying regional alterations that are common or different across varying types of dementia, hindering the use of the proposed neuroimaging markers in clinics.

While the small sample size of the test dataset can be considered a limitation, it is important to note that the purpose of the current work was not to distinguish which underlying causes of dementia closely resembled an AD-like pattern of metabolism. On the contrary, the present study instead highlights that the MAD algorithm in particular can readily distinguish between dementia and non-dementia cases. Further study with larger sample sizes is required to identify if hypometabolism or activity in specific regions is shared among all or most causes of dementia, as well as which regions are unique to specific diseases.

Another limitation was that the follow-up term after the FDG-PET scans was relatively short in all groups. A longer follow-up period would allow us to have a better understanding of the prediction outcomes in clinical settings. Finally, due to the retrospective nature of this study, the clinical variables were not available for all participants, restricting any systemic correlation analysis with imaging-based scores in different test groups.

## Figures and Tables

**Table 1:** Summary of symptoms and affected neuroanatomy of common dementia diagnoses.

Diagnosis		Symptoms	Affected Neuroanatomy
**Alzheimer’s disease (AD)**		Progressive episodic and semantic memory loss is the first and major symptom. Executive dysfunction, impaired judgement, changes in mood, personality, and behaviour, and language deficits (aphasia) are seen as the disease progresses [12,13]. Visuospatial deficits are also observed, but tend to be less severe than other cognitive symptoms [13].	Degeneration of medial temporal lobe structures (hippocampus, entorhinal cortex) [13]. Hypometabolism in limbic system (posterior cingulate and parahippocampal gyri), precuneus, posterior parietal cortex, and bilateral middle and inferior temporal gyri [14,15]. Prefrontal association cortex, temporoparietal, and posterior cingulate regions are associated with dementia severity [16].
**Atypical AD**	**Posterior cortical atrophy (PCA)**	Subtle vision problems that worsen over time including facial/object agnosias and alexia, difficulty with spatial awareness and judging distance. Later stages are likely to include memory loss, confusion, and communication difficulties. Most often (but not always) associated with AD [13,17].	Hypometabolism in striatum, lateral occipital lobe, inferior parietal lobe, lingual gyrus, posterior cingulate, cuneus, precuneus, supramarginal gyrus, and thalamus [18].
	**Frontal variant AD (fvAD)**	Early memory complaints and semantic language impairment. Irritability, delusions, and movement disorders (myoclonus) are also common [19].	Symmetrical degeneration of temporal lobes, with additional atrophy in frontal lobes, posterior corpus callosum and perisylvian region [19].
**Dementia with Lewy bodies**		Deficits in attention and visuospatial and executive functions, poor regulation of bodily functions, visual hallucinations, movement disorders (e.g., Parkinsonism), [1].	Hypometabolism in occipital cortex (primary visual cortex, Brodmann areas 17–19), temporal and parietal cerebral cortices [10,20].
**Frontotemporal lobar degeneration (FTLD)**	**FTLD (generally)**	Gradual and progressive deficits in behaviour (bvFTLD), language (PPA), or movement. Memory is often spared [21].	Hypometabolism in ventromedial and anterior frontal lobe, ventral temporal, and medial thalamic regions [22,23].
	**Behavioural variant FTLD, bvFTLD**	Late memory complaints. Language impairment (socioemotional aspects and phonemic fluency). Changes in personality and inappropriate behaviour, compulsivity, difficulty planning and organizing, apathy, anhedonia, lack of sympathy/empathy [1,2,19].	Symmetric or asymmetric frontotemporal cortex degeneration [19,21].
**Progressive supranuclear palsy, PSP**		Difficulty with rigidity, walking, postural instability, and eye movements. Executive dysfunction (difficulty concentrating, problem-solving, decision-making, and planning), and language deficits are common (depending on subtype) while memory is often spared [2,24].	Tauopathy with accumulation/degeneration in basal ganglia, diencephalon, and brainstem [24]. Hypometabolism in insular cortex, lateral and midline frontal cortex, cerebellum, brainstem, and caudate nucleus [25].
**Progressive primary aphasia (PPA)**	**PPA (generally)**	Difficulty with language most prominent feature that impairs daily living [2,26].	Hypometabolism in left parietal and posterolateral temporal lobes [9].
	**Semantic dementia**	Speech is fluent but understanding of word meaning is impaired. Prosody and syntax are spared while emotionality may be impaired [21,26,27].	Anterior temporal cortex atrophy, hypoperfusion or hypometabolism [26].
	**Progressive nonfluent aphasia**	Speaking becomes effortful, motor-related speech deficits and loss of grammar are evident while word knowledge is spared [21,26].	Left posterior fronto-insular atrophy, hypoperfusion or hypometabolism [26].
	**Logopenic aphasia**	Speech becomes slow and hesitant, finding the correct word becomes difficult, and sentence repetition is difficult [21,26,28].	Left posterior perisylvian or parietal atrophy, hypoperfusion or hypometabolism [26]. These patients sometimes present with AD at autopsy, but results have been controversial [2,13]
**Vascular dementia**		Varies, depending on location of stroke/lesion [1].	Hypometabolism and lesions in focal cortical and subcortical regions [29].
**Mixed dementia**		Dementia attributed to more than one cause, as such, symptoms vary as per other diagnoses.	Depends on diagnoses.
**Mild cognitive** **impairment ^1^**		Cognitive function worse than normal for the individual, but not causing interruption to daily living [1]	Posterior cingulate, inferior parietal lobe, and precuneus [9].
**Primary psychiatric disorder ^1^**		Various, depending on the diagnosis. Memory complaints, depressive or anxious mood, altered sleep cycles, slowed processing speeds, executive dysfunction, difficulty concentrating, worry, irritability, fatigue, muscle tension [30,31].	Increased activity in amygdala, altered activity in anterior cingulate cortex and hippocampus, and reduced activity in prefrontal cortex, [32,33].

^1^ Mild cognitive impairment (MCI) and primary psychiatric disorder are not classified as dementia although MCI can develop into dementia over time, and primary psychiatric disorders can mimic dementia symptoms.

**Table 2:** Demographic data with respect to dementia status (*n* = 47).

Category	Clinical Diagnosis	*n*	Mean Age (SD)	Male: Female	Average Follow-Up Duration (Months)
Dementia (*n* = 27)	Undifferentiated dementia	4	58.50 (4.72)	2:2	26.00 (4.24)^4^
	Alzheimer’s disease	5	61.00 (12.55)	2:3	29.80 (29.68)
	Posterior cortical atrophy	1	55.00 (0.00)	1:0	4.00 (0.00)
	Atypical Alzheimer’s disease, frontal	1	64.00 (0.00)	0:1	5.00 (0.00)
	Dementia with Lewy bodies	1	67.00 (0.00)	1:0	1.00 (0.00)
	Frontotemporal lobar degeneration ^1^	3	67.00 (9.85)	2:1	0.00 (0.00) ^4^
	Progressive supranuclear palsy, PSP	2	62.00 (1.41)	2:0	7.00 (1.41)
	Primary progressive aphasia ^2^	6	62.66 (7.60)	5:1	11.80 (8.04) ^5^
	Mixed dementia (vascular + AD) ^3^	4	70.50 (7.41)	3:1	13.75 (14.08)
Non-Dementia (*n* = 20)	Mild cognitive impairment	8	58.25 (9.13)	7:1	18.75 (18.96) ^4^
	Primary psychiatric disorder	3	56.00 (6.55)	2:1	50.00 (0.00) ^4^
	Cognitively healthy	9	54.0 (12.13)	3:6	8.11 (8.87)

^1^ Behavioural variant. ^2^ Participants with non-fluent, semantic, and logopenic primary progressive aphasia were grouped together. ^3^ In the present sample, mixed dementia is the coexistence of cerebrovascular disease and AD. ^4^ Two participants did not return for follow-up. ^5^ One participant did not return for follow-up.

**Table 3:** The proportion of each group that received a positive classification of AD from the SVM-ISDA classifier.

Category	Clinical Diagnosis	MAD
Dementia	Undifferentiated dementia	2/4
	Alzheimer’s disease	4/5
	Posterior cortical atrophy	1/1
	Atypical Alzheimer’s disease (frontal variant)	0/1
	Dementia with Lewy bodies	1/1
	Frontotemporal lobar degeneration (behavioural variant)	2/3
	Progressive supranuclear palsy	1/2
	Primary progressive aphasia	5/6
	Mixed dementia (vascular + AD)	2/4
	Total	18/27
Non-Dementia	Mild cognitive impairment	1/8
	Primary psychiatric disorder	0/3
	Cognitively healthy	1/9
	Total	2/20

MAD: Machine learning-based Alzheimer’s disease Designation algorithm.

## Data Availability

The MAD algorithm is available via the corresponding author’s lab website (https://www.kolabneuro.com/; accessed on 1 March 2019).
